# Engaging Virtually: Assessing the Impact of Schwartz Rounds in the Digital Era

**DOI:** 10.7759/cureus.78153

**Published:** 2025-01-28

**Authors:** William C Lippert, Cara J Haberman, Rebecca Omlor, Tina M Lovings, Gregory Russell, Maryjo Cleveland, Shannon G Hanson, F. Keith Stirewalt, Alexandra Godfrey

**Affiliations:** 1 Internal Medicine, Wake Forest University School of Medicine, Winston-Salem, USA; 2 Pediatric Medicine, Wake Forest University School of Medicine, Winston-Salem, USA; 3 Palliative Care, Wake Forest University School of Medicine, Winston-Salem, USA; 4 Respiratory Care, Atrium Health Wake Forest Baptist Medical Center, Winston-Salem, USA; 5 Biostatistics and Data Science, Wake Forest University School of Medicine, Winston-Salem, USA; 6 Geriatrics, Wake Forest University School of Medicine, Winston-Salem, USA; 7 Neonatology, Wake Forest University School of Medicine, Winston-Salem, USA; 8 Clinical Medicine, Wake Forest University School of Medicine, Winston-Salem, USA; 9 Emergency Medicine, Atrium Health Wake Forest Baptist Lexington Medical Center, Lexington, USA

**Keywords:** healthcare culture, humanizing healthcare, interprofessional communication, reflective practice, virtual schwartz rounds

## Abstract

Burnout and emotional strain among healthcare professionals are prevalent issues, impacting their personal wellbeing and affecting the quality of care they provide to patients. The Schwartz Center for Compassionate Healthcare (Boston, Massachusetts, United States) attempted to address some of these challenges by creating Schwartz Rounds®, which is a platform for caregivers to openly discuss the social and emotional aspects of their work. Our study evaluates the role played by virtual Schwartz Rounds in supporting healthcare workers during the COVID-19 pandemic.

Between April 2021 and November 2023, 28 virtual Schwartz Rounds sessions were hosted with a total recorded attendance of 2664. A total of 557 participants submitted completed survey responses and these were overwhelmingly positive, including new insights into colleagues' and patients' perspectives, reduced feelings of isolation, and increased openness in discussing patient care. Facilitators received high praise and nearly all attendees expressed their intent to attend future sessions. While the offering of incentives (e.g., Continuing Medical Education (CME) credit and raffle prizes) showed no significant impact on attendance or survey completion, the “challenge-focused” sessions were associated with higher attendance. Overall, our findings support virtual Schwartz Rounds as a way to promote staff well-being and collaboration. Future studies should be conducted at other institutions to assess the generalizability of our findings.

## Introduction

Careers in healthcare are inherently stressful and emotionally difficult. The prevalence of burnout among healthcare workers is estimated to be around 50% [1-3] and the prevalence of depression is around 30% [4-6]. Factors associated with high rates of burnout include patient-facing roles, perceived lack of safety, workers' own health problems, and poor work-life quality [7,8].

The Schwartz Center for Compassionate Healthcare (Boston, Massachusetts, United States) was founded on the idea of supporting the capacity for compassion amongst caregivers and healthcare workers [9]. Schwartz Rounds® is the specific arm of Schwartz programming that takes place within many healthcare institutions worldwide. The rounds are a regularly scheduled time for healthcare workers to “openly and honestly discuss the social and emotional issues they face in caring for patients and families” [9]. These sessions each center on a topic chosen by the planning committee and generally start with a multidisciplinary panel of speakers sharing stories related to the topic. The session facilitator then opens the conversation to the wider audience for further discussion and sharing.

The Schwartz Rounds program has been found to help participants gain a better perspective of both patients [10,11] and co-workers [12], decrease isolation, and share emotions more openly [12]. They have also been shown to improve attendees’ psychological well-being [13]. Since conception, a major aim of the Schwartz Rounds program has been to bring people together in a shared space [9]. Prior to 2020, it was recommended that lunch be provided during sessions in order to increase connection between participants. However, the COVID-19 pandemic in 2020 made gathering in-person and sharing food impossible.

While burnout has been evident in medicine for some time, the need for addressing this became more apparent during the COVID-19 pandemic. Hospital chief executive officers (CEOs) began to focus on physician wellness, and organizations such as the American College of Physicians expanded online support for physician well-being through Continuing Medical Education (CME) activities, TED talks (TED Conferences, LLC, New York City, New York, United States), podcasts, and tool kits [14]. Many, if not most of these activities, are solitary in nature such as exercise or meditation. Schwartz Rounds provides an alternative to this type of activity in that it is a collective experience that promotes the sharing of common emotions in a safe space. Ng et al. report the benefit of “realizing our humanity,” which allows the refocusing on altruism, connection, and compassion [15]. In this sense, Schwartz Rounds functions as a support group for those in attendance.

The institutional Schwartz Rounds program at Atrium Health Wake Forest Baptist (AHWFB) began in December 2018 in the children’s hospital and in April 2021 in the rest of the hospital. These programs merged in September 2022. Collectively, we both experienced switching to virtual programming and starting a virtual program. The initial funding for Schwartz Rounds at AHWFB was provided through the office of Dr. Julie Freischlag, then CEO of Wake Forest Baptist. After the initial funding, the Schwartz Rounds team was charged with developing future funding sources. The financial needs of Schwartz Rounds are minimized in two ways. All planning team members volunteer their efforts, participating without apportioned remuneration from AHWFB, and the annual costs of membership are covered by various departments.

At AHWFB, the intent is to appeal to as wide an audience as possible. The planning committee consists of physicians, nurses, pharmacists, physician assistants, chaplains, respiratory therapists, and administrators. Panelists are recruited from all departments that make up a hospital including providers, therapists, security, support staff, chaplaincy, and others. Because of this purposeful approach, the audience looks like the hospital, with the limitation of day shift staff only. The planning team meets weekly. No meeting takes place during weeks when Schwartz Rounds are presented. The planning team agenda focuses on the logistics of the upcoming Rounds, analysis of feedback on the immediately preceding rounds, and exploration of further topics.

The purpose of this study was to determine if virtual Schwartz Rounds met the intended goal of supporting healthcare workers, building connections, and maintaining the capacity for compassion.

## Materials and methods

This study was approved by the Wake Forest University School of Medicine Institutional Review Board under IRB Exempt status (IRB#: 00104182), as the study presented no more than minimal risk.

From April 2021 through September 2022, the Schwartz Rounds data were based solely on non-pediatric patients. After merging with Pediatric Schwartz Rounds in October 2022, data collection continued through the final observation period in November 2023. A total of 28 sessions were conducted: eight in the initial year, followed by 10 sessions each in 2022 and 2023. Attendance data, collected via the Webex platform (Cisco Systems, Inc., San Jose, California, United States), was available for all sessions, as there was no in-person option for attendance. 

To promote Schwartz Rounds sessions, multiple avenues were employed, including personal email invitations, invitations placed on the calendar on the hospital's internal page (intranet), session-specific flyers posted around the medical center, notices in *BestHealth/LiveWell* (the institution’s quarterly newsletter), and via advertisements on monitors throughout the hospital. To assess attendees’ opinions on each session, a survey was available at the end of each Schwartz Rounds session (see Appendices) and collected using REDCap electronic data capture tools hosted at Wake Forest University School of Medicine [16,17]. This survey was originally created by the Schwartz Center for Compassionate Healthcare for use during each Schwartz Rounds session [9]. Any survey that was not fully completed was excluded from the study.

With the data obtained from the survey responses and the attendance, the Schwartz Rounds were also categorized based on content offered within the session, whether the round was topic- or case-based, and whether raffle prizes were offered. Additionally, some sessions provided an opportunity for CME credits; another factor assessed included the focus of the Round, with the focal point defined in one of two ways: “positive-focused” or “challenge-focused.” The former involved themes that might be uplifting, providing positive emotions including happiness, hope, and optimism. For example, the November 2022 session centered on “Things that I am thankful for: gifts from my patients” in which the panelists shared positive stories about gifts they received from patients. Incorporating challenging themes that might be difficult to discuss (and demand more courage to do so) was also important as we wanted our series to be broad-reaching. Sessions such as the one in August 2021 on “Coping with medical error,” in which the panelists shared stories in which a medical error was made, were included with different healthcare workers discussing their experiences with medical error.

Data analysis included summary statistics (means and standard deviations for continuous measures and frequencies and proportions for categorical variables). The Wilcoxon Two-Sample Test (or Rank-Sum Test) was used to assess differences in attendance based on the session characteristics listed in the above paragraph with p-values < 0.05 assumed to be statistically significant. SAS version 9.4 (SAS Institute Inc., Cary, North Carolina, United States) was used for all statistical analyses.

## Results

Attendance and survey completion

Between April 2021 and December 2023, a total of 2664 participants attended the 28 Schwartz Rounds sessions (average number of participants per session was 95, standard deviation of 37). Of the 2664 participants, a total of 557 participants submitted completed survey evaluations. Of these, 189 identified themselves as a “nurse,” 174 identified themselves as “other profession,” 86 identified themselves as a “doctor,” 43 identified themselves as a “chaplain,” 33 identified themselves as “respiratory therapist,” 23 identified themselves as a “social worker,” seven identified themselves as a “physical and occupational therapist,” one identified themselves as “speech-language pathologist,” and one as “no response.” The proportion of attendees that completed the survey was highest in May 2021 (60.5%; 23/38) and lowest in November 2022 (9.1%; 4/44).

Responses

Notification of the Sessions

Based on the survey questions outlined, 74.3% (414/557) of the survey respondents reported that they heard about Schwartz Rounds from an “email invitation” and 16.5% (92/557) by “word of mouth.”

Communication and Experience with Co-workers, Patients, and Patient Families

Of the survey respondents, 97.1% (541/557) reported that the Schwartz Rounds session gave them “new insights into perspectives and experiences of their co-workers,” 92.3% (514/557) reported that the Schwartz Rounds sessions gave them “new insights into perspectives and experiences of patients and/or families,” 85.3% (475/557) reported that the Schwartz Rounds session made them “feel less isolated in their work with patients,” and 89.3% (497/557) reported that the Schwartz Rounds session made them “feel more open to expressing thoughts, questions and feelings about patient care with colleagues.”

“Hearing the frustrations and triumphs from other healthcare professionals gives me a sense of less isolation.” ~ survey respondent, May 2023

Evaluating the Facilitators and Overall Sessions

Of the survey respondents, 98% (546/557) reported that the Schwartz Rounds session was “well-facilitated,” 97.3% (542/557) reported that they “plan to attend Schwartz Rounds again,” 99.1% (552/557) rated the Schwartz Rounds as either “excellent” or “good” with 83.7% (466/557) being “excellent” and 15.4% (86/557) being “good.”

"Extremely well-facilitated" ~ survey respondent, June 21, 2022

"Best session and so needed. Amazing and so conflicting. Great facilitation!" ~ survey respondent, September 22, 2022

Effect of Various Factors on Attendance

Table 1** **includes the demographic data for each Schwartz Rounds session, including attendance, session type (topic-based or case-based), theme type (challenge-focused or positive-focused), and whether CME credits or raffle prizes were offered. There was an average of 95 participants at each session with a range of 35-168 participants. An average of 19 surveys were completed after each session (range: 4-49) with the survey completion rate based on attendance for the session being 20.9% (range: 9-60%). Attendance at Schwartz Rounds sessions was significantly higher after the merger of the adult hospital and pediatric hospital in October 2022 (p=0.008); however, the response rate for survey completion was significantly lower (p=0.003). The session type (topic-based or case-based) of the Schwartz Rounds session had no significant association with attendance (p=0.180) or the response rate for survey completion (p=0.710). The theme type of the Schwartz Rounds session had a significant association with attendance when the theme was challenge-focused (p=0.012). However, there was no significant association with the response rate for survey completion based on the theme type of the Schwartz Rounds session (p=0.067). The offering of a raffle prize by completing the survey was not associated with a higher response rate for survey completion (p=0.260) nor higher attendance of the Schwartz Rounds session (p=0.590). CME being offered for the Schwartz Rounds session was not associated with higher attendance (p=0.170) nor a higher response rate for survey completion (p=0.430).

**Table 1:** Characteristics of each Schwartz Round session organized by month. Note: Merger with Pediatric Schwartz Rounds occurred in October 2022. Schwartz Rounds sessions not held in December 2021, May 2022, December 2022, July 2023, and December 2023. CME: Continuing Medical Education

Month	Attendance	Number of Evaluations Completed	CME Offered?	Theme	Session Type	Raffle Prize Offered?
April 2021	94	49	No	Challenge-focused	Topic-Based	No
May 2021	38	23	No	Positive-focused	Topic-Based	No
June 2021	35	16	No	Challenge-focused	Topic-Based	No
July 2021	35	13	No	Positive-focused	Topic-Based	No
August 2021	72	10	Yes	Challenge-focused	Topic-Based	No
September 2021	36	12	Yes	Positive-focused	Topic-Based	Yes
October 2021	64	30	Yes	Challenge-focused	Topic-Based	Yes
November 2021	55	19	Yes	Positive-focused	Topic-Based	Yes
December 2021						
January 2022	76	13	Yes	Challenge-focused	Topic-Based	Yes
February 2022	137	25	Yes	Challenge-focused	Topic-Based	Yes
March 2022	115	24	Yes	Challenge-focused	Topic-Based	Yes
April 2022	93	12	Yes	Positive-focused	Topic-Based	Yes
May 2022						
June 2022	94	22	No	Challenge-focused	Topic-Based	Yes
July 2022	101	11	No	Challenge-focused	Topic-Based	Yes
August 2022	96	20	No	Challenge-focused	Case-Based	Yes
September 2022	136	37	No	Challenge-focused	Topic-Based	Yes
October 2022	134	43	No	Challenge-focused	Topic-Based	No
November 2022	44	4	No	Positive-focused	Topic-Based	No
December 2022						
January 2023	98	9	No	Challenge-focused	Topic-Based	No
February 2023	102	17	No	Positive-focused	Topic-Based	No
March 2023	130	17	No	Challenge-focused	Topic-Based	No
April 2023	94	10	No	Challenge-focused	Topic-Based	No
May 2023	145	27	No	Challenge-focused	Case-Based	No
June 2023	127	20	No	Challenge-focused	Topic-Based	No
July 2023						
August 2023	107	18	No	Challenge-focused	Topic-Based	No
September 2023	123	15	No	Challenge-focused	Case-Based	No
October 2023	168	25	No	Challenge-focused	Topic-Based	No
November 2023	115	16	No	Positive-Focused	Topic-Based	No
December 2023						
Total	2664	557	No: 20 Yes: 8	Challenge-Focused: 20 Positive-Focused: 8	Topic-based: 25 Case-based: 3	No: 17 Yes: 11

## Discussion

To the best of our knowledge, this is the first study to evaluate the impact of virtual Schwartz Rounds sessions. Our findings provide valuable insights into the engagement and perceived value of virtual Schwartz Rounds amongst healthcare professionals. Overall, we believe that the virtual environment of Schwartz Rounds can be just as impactful for staff as in-person Schwartz Rounds sessions, particularly in the current digital era. Overwhelmingly the attendees reported that it improved communication and experience with co-workers, patients, and patients’ families. These findings are consistent with other studies of in-person Schwartz Rounds sessions [10-12,18-22]. Moreover, our virtual Schwartz Rounds sessions were reported to mitigate feelings of isolation and create a supportive environment for healthcare professionals to share experiences and emotions openly, which is similar to findings in other studies of in-person Schwartz Rounds sessions [12,15,18,21,23].

We also found it interesting that in terms of notification regarding each Schwartz Rounds session, personal email invitations were the most effective channel based on 74.3% of our survey respondents. While word-of-mouth also was a notable contributor, efforts to enhance digital outreach and communication strategies could further improve attendance rates at our institution and at other institutions as well.

Our virtual Schwartz Rounds sessions were well attended with an average of 95 participants per session. This was on par with attendance figures reported in the literature with in-person attendance from other studies [10,12,18,22,24]. It should be noted that we did not provide food as part of Schwartz Rounds sessions, which was the recommendation for these in-person sessions. We all know that free lunch can often be an attraction to conferences. With that said, we still held our sessions during the lunch hour (12:00-1:00 pm) in order to maximize attendance, and let people know they were free to eat, as this seemingly is the best time to hold Schwartz Rounds sessions per Silke et al.’s findings [22]. Interestingly, the other tactics we employed to help boost attendance, and thought would help bolster attendance, seemingly had no effect. For example, the offering of CME credits for the session was not associated with a higher attendance (p=0.170) and offering a raffle prize (e.g., a tumbler) for completing the survey at the end of the session too had similar results (p=0.590). These findings suggest that the intrinsic value of the sessions, rather than extrinsic rewards, drives engagement among participants. Also, due to the administrative burden associated with CME and raffle prizes, our Schwartz Rounds Planning Committee decided not to pursue these options at our future sessions. However, we did note that the challenge-focused themed Schwartz Rounds were associated with significantly higher attendance than positivity-focused themed Schwartz Rounds (p=0.012). We found this to be an interesting point, but still think there is value in having a diversity of themes for the sessions and do not advocate for strictly challenge-focused themed Schwartz Rounds sessions in our or other institutions’ Schwartz Rounds curriculum.

In terms of survey completion, there were on average 19 surveys completed per session (range: 4-49 surveys completed per session). This was slightly lower than previously reported by Flanagan et al. who had an average of 33 surveys completed per session [20]. We employed tactics to boost our survey response rate, similar to attendance, but they seemingly had no effect. Selecting a random winner for a raffle prize for completing the survey was not associated with a higher response completion rate (p=0.260), nor was the offering of CME (p=0.430), nor the theme (positive-focused vs. challenge-focused) of the Schwartz Rounds session (p=0.710). However, we did note an interesting finding that the response rate for survey completion was significantly lower (p=0.003) when we merged the adult hospital and pediatric hospital sessions in October 2022, despite attendance being significantly higher after the merger (p=0.008). We hypothesize that attendees who attended consecutive Schwartz Rounds sessions likely completed them the first session that they attended, but stopped completing them after subsequent sessions they attended.

We also found it interesting that the facilitation of the Schwartz Rounds sessions was also a likely key factor contributing to our success with 98% of survey respondents acknowledging effective facilitation. This finding underscores the importance of skilled facilitators in guiding these Schwartz Rounds sessions and ensuring that they maintain a safe and respectful atmosphere conducive to reflection and learning.

The results of the study must be interpreted within the context of its design and therefore had limitations. The attendance data is likely higher than what is reported because this information was extracted from the Webex platform. There were often multiple people seated at the same computer or seated in a conference room, but in those situations, it only counted as one attendee. The survey that was used in this study is not validated but is the standard survey distributed from the Schwartz Center for Compassionate Healthcare [9]. Many of the other studies used as a comparison used the same survey.

Overall, our study validates that virtual Schwartz Rounds sessions can be just as impactful in promoting emotional well-being among healthcare professionals as in-person Schwartz Rounds sessions. Moving forward, efforts to enhance communication strategies, optimize session formats, and leverage digital platforms effectively can further maximize the impact of Schwartz Rounds in supporting the needs of healthcare workers.

## Conclusions

This is the first study to our knowledge assessing the impact of virtual Schwartz Rounds sessions. The virtual environment provides a positive impact for those who attend Schwartz Rounds, which is consistent with those who have attended in-person. The offering of raffle prizes and CME had no effects on attendance or survey completion. However, the challenge-focused themed sessions were associated with higher attendance, although we suggest a diversity of themes for the sessions. Further studies need to be conducted to determine generalizability on the impact of virtual Schwartz Rounds in this digital era.

## Appendices

Survey questionnaire

This is a copy of the RedCap evaluation that was shared with the audience at the end of each session and completed voluntarily by attendees. This survey was adopted from the Schwartz Center for Compassionate Healthcare [9]. (*Please note that question 12 was only included in the evaluation from September 2021 through September 2022 in order to be randomly selected for a raffle prize.)

Schwartz Rounds Evaluation

Thank you for attending today's Schwartz Rounds session.

Please complete the following evaluation form. Your feedback is sincerely appreciated and helps the Planning Committee improve the program for future sessions.

1a. Check your professional affiliation:

 Chaplain

 Doctor

 Nurse

 Physical and Occupational Therapy

 Respiratory Therapy

 Speech-Language Pathology

 Social Worker

 Other Professional 

1b. If “Other Professional,” please specify __________________________________

2a. How did you hear about the Schwartz Rounds session today?

 Email invite

 Intranet

 Word of mouth

 Other

2b. If “Other,” Please specify __________________________________

In the following questions (3-9), please check the answer that best reflects your opinion about each of the following statements:

3. Today's discussion gave me new insights into the perspectives and experiences of my co-workers.

 No

 Yes

 Not sure

4. Today's discussion gave me new insights into the perspectives and experiences of patients and/or families.

 No

 Yes

 Not sure

5. As a result of this discussion I feel less isolated in my work with patients.

 No

 Yes

 Not sure

6. As a result of this discussion I feel more open to expressing thoughts, questions and feelings about patient care with colleagues.

 No

 Yes

 Not sure

7. I plan to attend Schwartz Center Rounds again.

 No

 Yes

 Not sure

8. Today's discussion was well-facilitated.

 No

 Yes

 Not sure

9. Please rate today's overall program:

 Poor

 Fair

 Neutral

 Good

 Excellent

10. Please describe a way that today's discussion will change how you relate to or communicate with patients and/or colleagues (if any):

__________________________________________________________________

11. Additional comments, including ideas for future topics or recommendations for future panelists:

__________________________________________________________________

*12. If you are interested in being included for a raffle prize, please include your email address here: __________________________________
